# Comparing saliva collection and DNA extraction methods for saliva-based microbiome profiling

**DOI:** 10.3389/fmicb.2026.1809075

**Published:** 2026-05-14

**Authors:** Oryan Agranyoni, Robert H. Yolken, Sara B. Johnson, Heather Volk, Sarven Sabunciyan

**Affiliations:** 1 Department of Pediatrics, Johns Hopkins School of Medicine, Baltimore, MD, United States; 2 Department of Population, Family and Reproductive Health, Johns Hopkins Bloomberg School of Public Health, Baltimore, MD, United States; 3 Wendy Klag Center for Autism and Developmental Disabilities, Johns Hopkins Bloomberg School of Public Health, Baltimore, MD, United States; 4 Department of Mental Health, Johns Hopkins Bloomberg School of Public Health, Baltimore, MD, United States

**Keywords:** 16s rDNA sequencing, collection and isolation kits, extraction kits, oral-microbiome, saliva

## Abstract

**Aims:**

Mounting preclinical evidence demonstrates the importance of the human microbiome in health and disease. Saliva presents a particularly appealing medium for microbiome research due to its non-invasive collection and the availability of extensive biobanked samples across various conditions. However, methodological challenges remain- particularly regarding sample storage and the variability introduced by different nucleic acid extraction kits, which can exhibit selective affinities for certain bacterial taxa. In this study, we systematically compared multiple saliva collection and DNA extraction methods to optimize protocols for 16S rRNA-based microbiome profiling. Our approach incorporated rigorous quality control measures, including the analysis of water controls, differential abundance testing, and correlation analyses across groups, to identify the most reliable and reproducible methods for salivary microbiome characterization.

**Methods and results:**

We compared four commercially available kits for at-home saliva collection to determine their effectiveness at preserving the salivary microbiome following 1 week of storage at room temperature (RT). We also compared three commercially available DNA extraction kits marketed for salivary microbiome characterization. We discovered that the DNA extraction kit used significantly impacted the microbiome composition. One week of incubation in the preservative solution shifted the bacterial composition of the saliva. Additionally, we demonstrated that contaminants in the environment and kits reagents may be increased during the incubation period, posing a significant challenge.

**Conclusion:**

Our work demonstrates the feasibility of, and provides a framework for, microbiome characterization in saliva that applies to clinical and population-based studies. Our findings indicate that saliva microbiome studies using different extraction kits may introduce systematic biases, which should be accounted for when comparing results across studies. Using the same nucleic acid collection and extraction kits in various experiments is essential for reproducibility due to their different affinity to specific bacteria and contamination rates.

## Introduction

In the last decade, studies have indicated that a balanced microbiome in multiple human tissues, including the oral and the gastrointestinal tracts (GI), is a key indicator of health (Lynch and Pedersen, 2016; Shreiner et al., 2015; Zarco et al., 2012). The human oral cavity possesses the second most abundant and diverse microbiome composition after the GI (Deo and Deshmukh, 2019), and it harbors numerous microorganisms, including bacteria, fungi, viruses, and protozoa (Deo and Deshmukh, 2019). While most microbiome studies focus on stool microbiome composition, altered oral microbiome has been observed in several diseases such as diabetes (Xiao et al., 2017), cancer (Chattopadhyay et al., 2019), endocarditis (Zhu et al., 2018), bacteremia (Han and Wang, 2013), autoimmune disease (Abe et al., 2020), and autism (Olsen and Hicks, 2020). Therefore, it is crucial to understand the oral microbiome composition and its variations under various disease conditions. The oral cavity possesses different niches, including the gingival sulcus, the tongue, the cheek, the hard and soft palates, the floor of the mouth, the throat, the saliva, and the teeth (Deo and Deshmukh, 2019). The microbial composition of each is unique. However, the bacterial composition of saliva includes bacteria detached from various niches in the oral cavity and resembles that of the tongue coating (Yamashita and Takeshita, 2017), making the saliva microbiome the representative of oral health. Saliva, a hypotonic solution of salivary acini, gingival crevicular fluid, and oral mucosal exudates, is a potential source for biological material such as biochemicals, DNA, RNA, proteins, and the microbiota. Saliva collection is non-invasive, and generally acceptable for most patient populations. Hence, saliva provides a non-invasive and convenient way to develop novel disease diagnosis tools (Zhang et al., 2016; Agranyoni et al., 2025).

Saliva collection in the field presents several challenges. Usually, sample collection and storage involve freezing of saliva, since room temperature storage may result in DNA degradation. However, freezing is not always feasible in the context of self-collection or collection in the field. A potential solution to this problem is the transport of saliva in a preservative liquid that prevents DNA degradation. Another challenge is collecting saliva from populations that cannot spontaneously provide a saliva sample via passive drool or other means (i.e., infants, young children, and individuals with developmental conditions). Using swabs (Verdon et al., 2014) to collect pooled saliva and storing specimens in a preservative liquid offers a potential solution for these storage and collection problems. However, little is known about how the composition of kits and swabs may impact composition of the microbiota.

While contamination in 16S rRNA gene sequencing workflows has been widely reported, less is understood about how combined methodological choices across sample collection, storage, and DNA extraction influence observed microbiome profiles under realistic conditions. In particular, most prior studies have evaluated individual components in isolation, rather than assessing how these factors interact within practical workflows (Kuffel et al., 2024). In addition, saliva-based microbiome studies often rely on swab-based collection methods, which are essential for populations unable to provide spontaneous saliva samples, yet these approaches have not been systematically evaluated alongside downstream processing steps (Deeley et al., 2016). Swab samples are commonly used in studies where participants self-collect samples, preserve them in stabilizing media, and mail them to researchers, enabling the efficient recruitment of large cohorts, including pediatric populations. The combined impact of swab-based collection and storage in preservative has not yet been systematically evaluated. Therefore, a comprehensive, integrative comparison of commonly used collection and extraction methods under real-world conditions remains lacking.

This study systematically compared methods for generating 16S microbiome data from saliva samples. We compared methods for collecting saliva and extracting DNA for 16S sequencing using four swab-based collection kits and three DNA column-based extraction kits. We demonstrate that factors including the choice of preservative, DNA extraction method, and contamination collectively influence the observed microbial composition of saliva, highlighting how interacting methodological choices can shape microbiome profiles and introduce systematic biases in downstream analyses.

## Materials and methods

### Ultra-pure water decontamination

To ensure the ultra-pure water (UPW) (Cat No. 351–161-671, Quality Biological, United States) was free of bacterial contamination, UV radiation was performed. UPW was divided into uncovered 2 mL aliquots and placed in Microprocessor-Controlled UV Crosslinkers (Spectroline, United States), energy dose 0.9999 J/cm2 for 10 min.

### Sample collection

Four different commercially available saliva collection kits were tested. Each included a swab and a collection tube with DNA preservative (a) ORACollect (Cat No. OC-175, DNA Genotek Inc., Canada), according to the manufacturer, the DNA stabilizing reagent inhibits the growth of bacteria from the time of sample collection to processing, and the DNA remains stable at ambient temperature for 1 year. (b) NORGEN BIOTEK (Cat No. 45681, NORGEN BIOTEK Corp., Canada), according to the manufacturer, this kit completely stabilizes microbiota profiles from the collection point, and the DNA preservation can stay at room temperature for over 2 years. (c) Omnigene.oral CORP (Cat No. OM-505, DNA Genotek Inc., Canada), according to the manufacturer, this kit provides a snapshot of the microbial profile at the time of collection, prevents microbial growth, and stabilizes RNA at room temperature and DNA up to 37 °C for 3 weeks. (d) ORAgene. DISCOVER (Cat No. OGR-675, DNA Genotek Inc., Canada), according to the manufacturer, the DNA has been stable for years at ambient temperature.

### Samples collected

For each collection method, we sampled UV-treated water for negative control (Water), and human pooled saliva (Saliva) (Cat No. 991-05-P-50, Lee Biosolutions, United States) from two different lots, following the manufacturer’s instruction. Sampling was performed in a laminar flow hood; we sampled 1.5 mL sterile tubes filled with saliva using the swab-based kits mentioned above. After sampling, all collection tubes were incubated for a week at room temperature (RT).

### DNA extraction

Three DNA extraction kits were tested according to manufacturer instructions: BIOCHAIN Saliva DNA Isolation Kit (Cat No. K5011050, BioChain Institute Inc., United States), NORGENE BIOTEK Saliva DNA Isolation Kit (Cat. RU45400, NORGENE BIOTEK, Canada), and QIAamp DNA Microbiome Kit (Cat No. 51704, QIAGEN, Germany). DNA concentrations were quantified using Qubit fluorometric quantification (Invitrogen, United States). Purified DNA samples were stored at −20 °C for further analysis.

### Library preparation

PCR was performed using Q5 High Fidelity 2X Master Mix (New England Biolabs, France) and primers targeting the V3–V4 region of the 16S rRNA gene with overhang sequences compatible with Illumina library preparation. The primer sequences were as follows (Klindworth et al., 2013): forward 5’-TCGTCGGCAGCGTCAGATGTGTATAAGAGACAGCCTACGGGNGGCWGCAG−3’ and reverse 5’-GTCTCGTGGGCTCGGAGATGTGTATAAGAGACAGGACTACHVGGGTATCTAATCC-3’. Following by gel electrophoresis 1.2% or 2.2% agarose (Cat No. 57023 and 57031, LONZA, Switzerland), marker was 50-1500 bp (Cat No. 57033, LONZA, Switzerland).

The library was prepared according to Illumina’s 16S Metagenomic Sequencing Library protocol with Nextera XT Index Kit v2 Set C, and Set D. Most saliva DNA samples were under 0.1 ng/ul, so adjustments were made to apply up to 10ul with a maximum of 5 ng DNA to the 1st Stage PCR. Library Quantification used Qubit dsDNA HS Assay for concentration and TapeStation D1000 ScreenTape for size. The final Library was normalized to 4 nM and pooled for sequencing.

### Bioinformatic processing

Raw paired-end FASTQ reads were processed and analyzed using the QIIME2 pipeline (version 2022.8) (Bolyen et al., 2019). Paired-end sequences were demultiplexed using the q2-demux plugin. Denoising, chimera removal, and amplicon sequence variant (ASV) inference were performed using DADA2 via the q2-dada2 plugin to improve taxonomic resolution (Callahan et al., 2016). Multiple sequence alignment of ASVs was conducted using MAFFT (Katoh et al., 2002), and a phylogenetic tree was constructed using FastTree2 through the q2-alignment and q2-phylogeny plugins, respectively (Price et al., 2010). Taxonomic classification was performed using the q2-feature-classifier, with feature sequences aligned against the Greengenes database (99% confidence) (Bokulich et al., 2018; McDonald et al., 2012).

To reduce potential contamination and spurious features, the feature table was filtered using the q2-feature-table plugin. First, features annotated as mitochondria or chloroplasts were removed. Next, features present in ≤10% of samples per group were filtered out, and features with a total relative frequency <0.001% across all samples were excluded.

Alpha diversity was calculated after rarefaction to an even sequencing depth (subsampling without replacement) to account for differences in sequencing depth across samples. Alpha diversity metrics included observed ASVs (richness), Shannon diversity (richness and evenness), and Faith’s phylogenetic diversity (PD) (Wagner et al., 2018). Alpha diversity was calculated after rarefaction to an even sequencing depth of 5,516 reads per sample. The non-swabbed Biochain water sample was excluded from this analysis due to its substantially lower sequencing depth, which would otherwise bias rarefaction. Beta diversity differences between groups were assessed using PERMANOVA based on Bray-Curtis distances with 999 permutations.

Due to the high prevalence of zero counts across ASVs, differential abundance analyses were performed using zero-inflated count models. Specifically, zero-inflated negative binomial (ZINB) models were applied using the R package zinbwave (Risso et al., 2018). Weights estimated by zinbwave were subsequently incorporated into differential abundance testing using DESeq2, which models count data while accounting for library size differences. Prior to ZINB and DESeq2 analyses, an additional filtering step was applied to retain features with a minimum total count of 500 reads across all samples. Principal component analysis (PCA) was performed using the plotPCA() function on variance-stabilized counts obtained via the varianceStabilizingTransformation() function in DESeq2 (Timmer et al., 2021; El Mouzan et al., 2022; Liao et al., 2022). All statistical analyses were conducted in R version 4.5.0 (Love et al., 2014).

## Results

### Comparison of sample collection and DNA extraction methods

**Figure 1:**
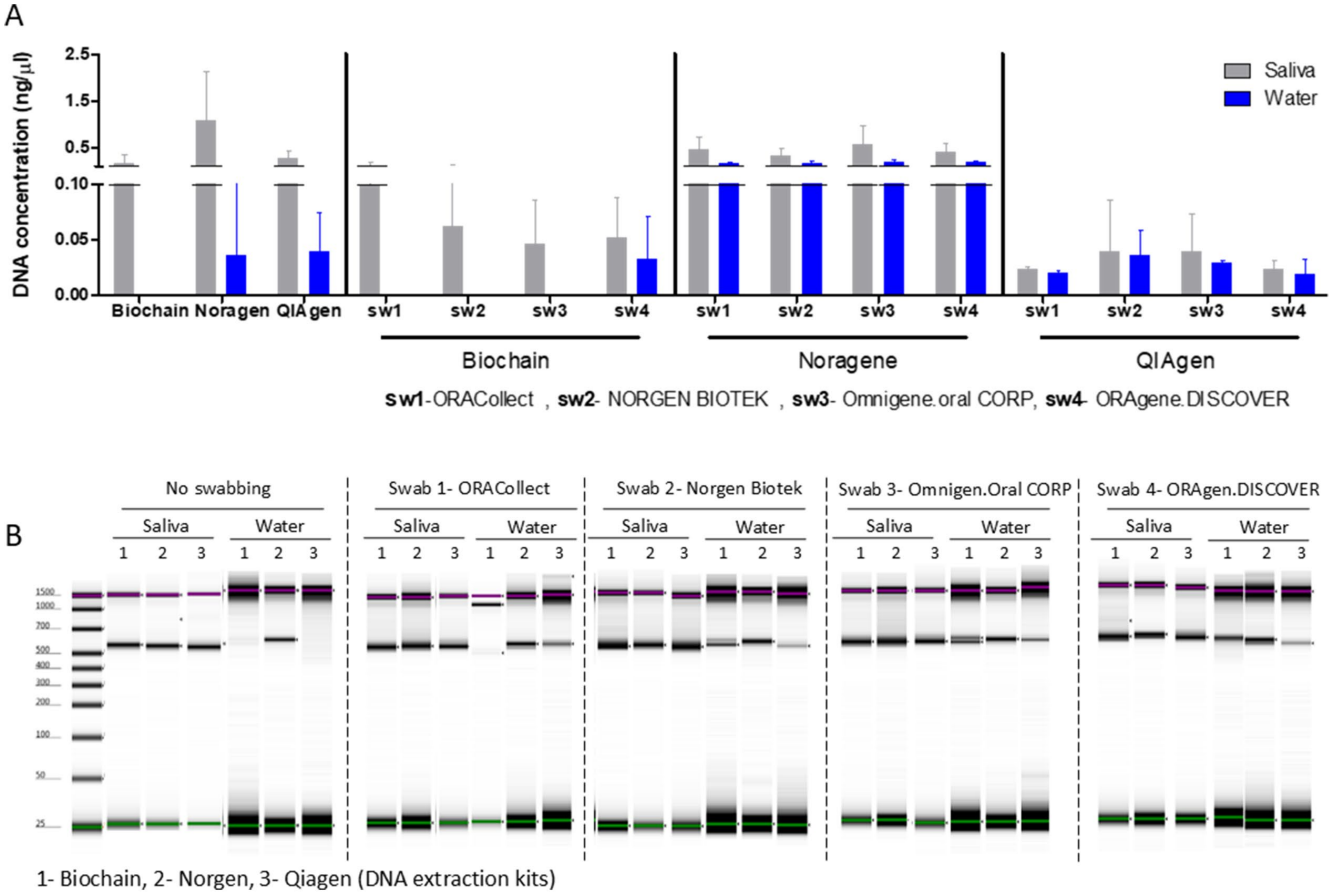
DNA concentration and 16S amplification gel. **(A)** DNA concentration of UV-treated samples and human pooled saliva with and without swabbing (*n* = 4 in each bar). **(B)** Amplicons of PCR amplification for the 16S v3-v4 rRNA gene from UV-treated samples and human pooled saliva with and without swabbing. This figure presents DNA yield and PCR-based validation of bacterial DNA across different collection and extraction conditions.

We compared kits for saliva collection followed by DNA extractions for bacterial composition analysis. We tested four different saliva collection kits and three different DNA extraction kits. We performed swab and non-swab sampling from human pooled saliva samples from two different lots (Saliva 1; Saliva 2), and from a UV-treated UPW samples (designated-water, divided into 1.5 mL Eppendorf’s, one for each swab and kit). For the swabbing procedure, the swab was inserted into each sample and then placed in the preservative reagent supplied in the sample collection kit (in a biological hood and sterile conditions). Then, after 1 week of incubation, we extracted DNA from the water (negative control, *n* = 4) and human pooled saliva (*n* = 4, 2 for each lot; Figure 1A), and quantified the total DNA extracted from each sample, using the Qubit fluorometric quantification instrument. The yield of DNA isolated by all extraction kits was low (<2.5 ng/μl Figure 1A). DNA was observed in the water samples extracted with the Norgen and Qiagen kits, with and without swabbing, and also in one sample extracted with the Biochain kit after swabbing, suggesting the presence of contaminants in the kit or in the swabs (bacterial or not). Norgen also had the highest DNA concentration after extraction from saliva samples (Figure 1A). This figure summarizes DNA yield and PCR validation across collection and extraction methods (Figure 1).

PCR amplification of the 16S rRNA gene was performed, and bands at ~500 bp indicate the presence of detectable bacterial DNA (Figure 1B). A visible band in the non-swabbed water control processed with the Norgen kit suggests the presence of bacterial DNA in kit reagents. After swabbing, visible bands were observed in all water samples (Figure 1B), indicating detectable levels of bacterial DNA, although low-level amplification below the detection limit of gel electrophoresis cannot be excluded. These observations were further supported by sequencing-based analyses (see below).

### Collection and extraction methods affect salivary bacterial composition measurements

To evaluate the collection and extraction kits’ quality in maintaining the accurate representation of the original sample of the salivary microbiome, we further analyzed the microbiome composition. Sequencing the V3–V4 regions of the 16S rRNA gene generated approximately 20.7 million sequences. The mean was 209,650 sequence reads per subject, and the filtered dataset contained 22,934 features covering 10 phyla, 14 classes, 22 orders, 31 families, 33 genera, and 20 species. The dominant phyla in the salivary microbiome across all cohorts were Firmicutes, Actinobacteria, Bacteroidetes, and Proteobacteria.

**Figure 2:**
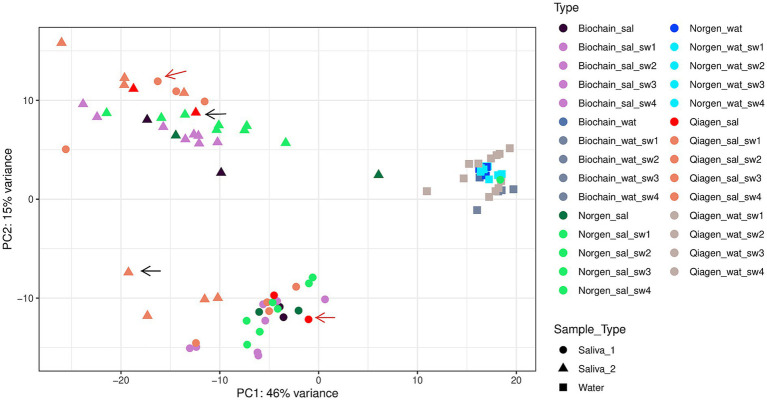
PCA plot of saliva and water samples with and without swabbing. Saliva 1, saliva 2, and water samples are clustered differently. The PCA was plotted from the DESeq2 object using R software. This figure shows overall clustering of samples across all experimental conditions, including differences in sample origin, extraction kit, and swabbing status.

PCA analysis was used to assess overall differences in microbial composition across all experimental conditions, including sample origin, extraction kit, and swabbing status (Figure 2). PCA analysis exhibits that water and saliva samples were clustered differently, regardless of the extraction and collection kits, as expected. Moreover, the saliva samples clustered differently within, so saliva 1 (circles) and 2 (triangles) clustered separately. Interestingly, the original samples extracted with the Norgen and Biochain kits clustered with the swabbed saliva samples extracted with the same kit, following 1 week of incubation. However, swabbed saliva samples extracted with the Qiagen kit did not cluster fully with their non-swabbed counterparts (Figure 2; see black and red arrows indicating samples that fell outside their expected cluster).

PERMANOVA analysis based on Bray-Curtis distances demonstrated that DNA extraction kit (*R*^2^ = 0.127, *p* = 0.001) and sample origin (saliva vs. water; *R*^2^ = 0.300, *p* = 0.001) significantly influenced overall microbial composition. In contrast, swabbing status did not have a significant effect (*R*^2^ = 0.012, *p* = 0.298). A combined model including all factors remained significant (*R*^2^ = 0.405, *p* = 0.001), indicating that both methodological and sample-related variables contribute to variation in microbiome profiles. PERMANOVA analysis comparing original and processed (swabbed) saliva samples did not reveal a significant difference in overall microbial composition (Bray–Curtis, *R*^2^ = 0.016, *p* = 0.438), In contrast, models including both processing and DNA extraction kit were significant (*R*^2^ = 0.112, *p* = 0.001), indicating that variation is primarily driven by differences between extraction kits rather than processing. Consistent with the clustering patterns observed in the ordination analysis.

**Figure 3:**
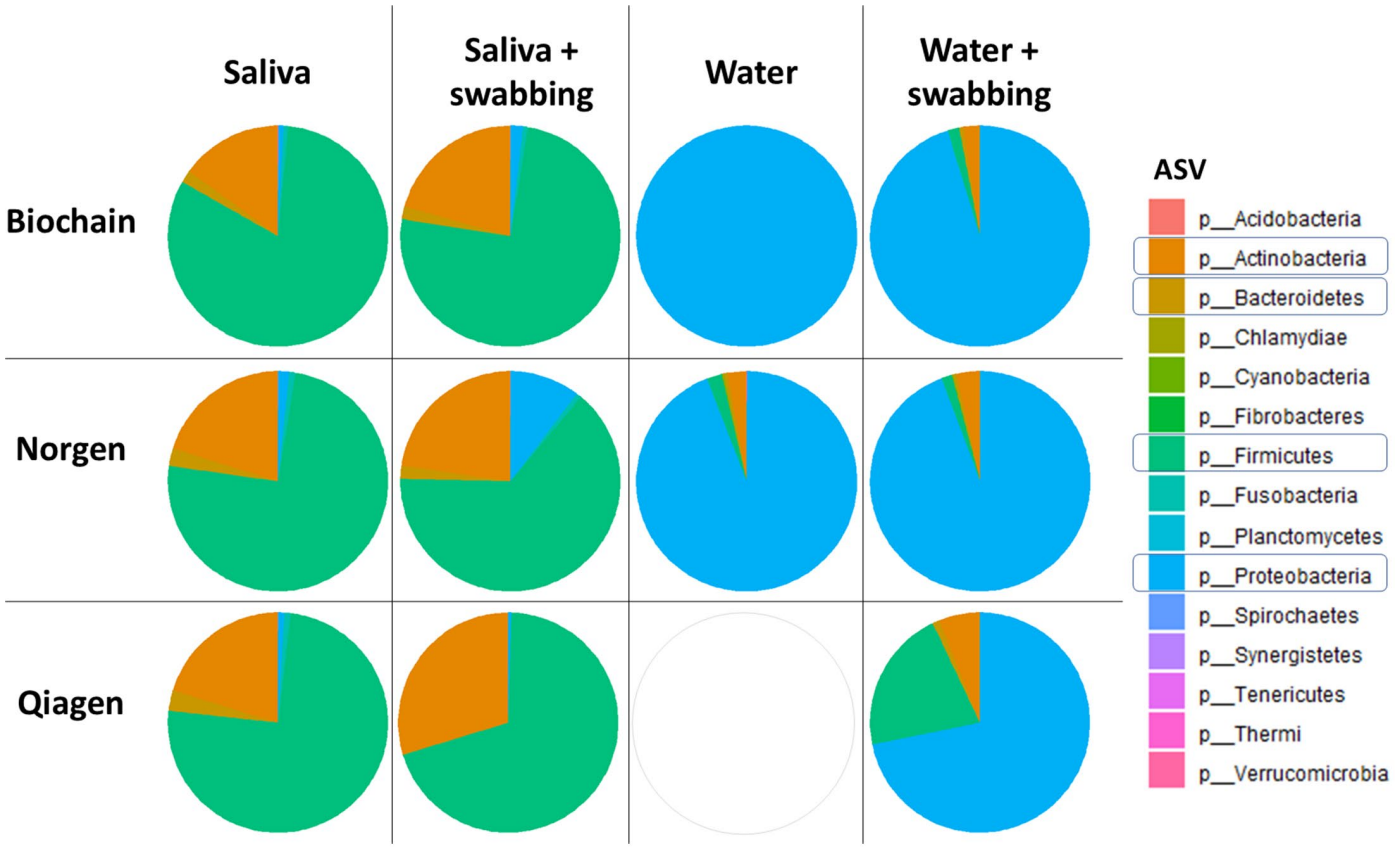
Phyla level composition of the salivary microbiome. Pie charts made with R software of the average microbiome composition of saliva or water samples with and without swabbing. This figure focuses on contamination profiles in water control samples across different kits and conditions.

To specifically assess contamination introduced by collection and extraction procedures, we analyzed water control samples (Figure 3). The primary contamination in the water samples, with or without swabbing, was due to *Proteobacteria* phyla (Figure 3). Moreover, swabbing and incubating the samples for 1 week prior to extraction, regardless of the extraction kit used, increased contamination levels of *Proteobacteria*, *Actinobacteria*, *Bacteroidetes*, and *Firmicutes* phyla (as seen in the water samples after swabbing, Figure 3). Interestingly, the samples extracted with the Norgen kit had the most significant addition of the *Actinobacteria* and *Proteobacteria* phyla, which we mainly attribute to the extraction kit contamination, as seen previously (Figure 1), while the water samples extracted with the Qiagen kit were contaminated with the *Firmicutes* phyla. Deeper characterization of the ASVs present in the water samples revealed that non-swabbed water samples extracted with a Biochain kit mainly consisted of *the Burkholderia* genus and that swabbed water samples consisted mainly of *Paracoccus*, *Stenotrophomonas*, and *Sphingomonas* genus, as well as *Alphaproteobacteria* Class, *Caulobacterales* order and *Proteobacteria* phyla. Water samples extracted with the Norgen kit mainly consisted of *Alphaproteobacteria* Class and *Burkholderia* genus with and without swabbing. In contrast, swabbed water samples extracted with a Qiagen kit consisted mainly of *Acetobacter*, *Staphylococcus*, *Lactobacillus*, and *Melissococcus* genus (Supplementary Figure 2S).

**Figure 4:**
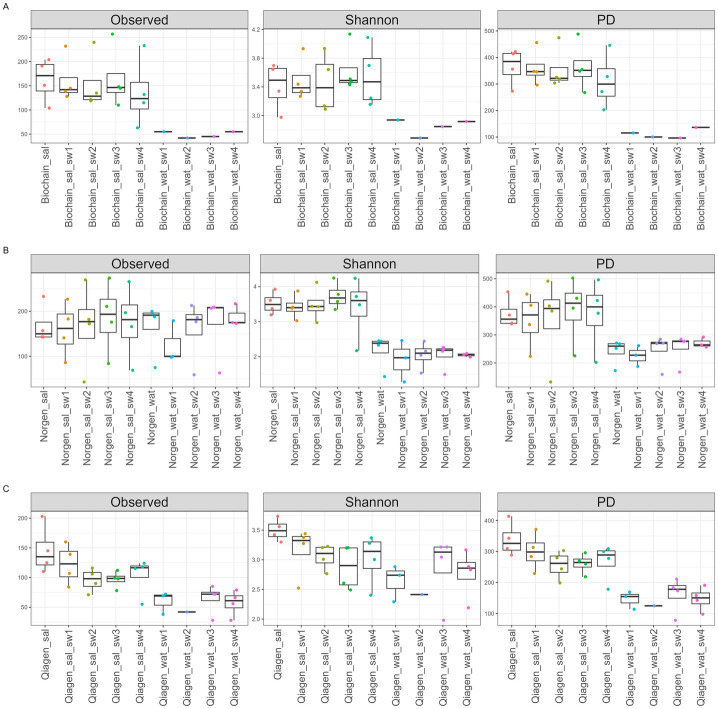
Alpha diversity of the salivary microbiome. Boxplots illustrating alpha diversity indices of observed ASVs, Shannon-Weiner index, and PD, the Faith’s phylogenetic diversity, in bacterial microbiomes of **(A)** bacterial 16S extracted with Biochain DNA extraction kit, **(B)** bacterial 16S extracted with Norgen DNA extraction kit, and **(C)** bacterial 16S extracted with Qiagen DNA extraction kit. Sal- saliva. SW- swab. Wat- water. This figure evaluates the effect of processing (swabbing and storage) on alpha diversity metrics.

To assess whether sample processing affected overall microbial diversity in saliva samples, we calculated observed richness, Shannon diversity, and Faith’s phylogenetic diversity after rarefaction to an even sequencing depth of 5,516 reads per sample (Figure 4). Overall, similar diversity values were observed between original and processed saliva samples. Across all extraction kits (Biochain, Norgen, and Qiagen), saliva samples collected using different swabbing methods showed comparable richness, evenness, and phylogenetic diversity relative to the corresponding original saliva samples processed without swabbing, with no statistically significant differences detected (*p* > 0.05; Figure 4).

**Figure 5:**
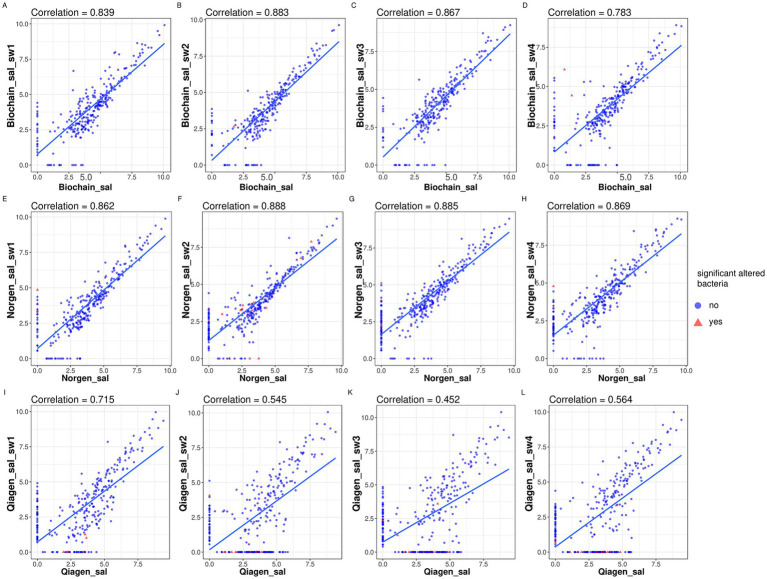
Correlational analysis of microbiome profiles from swabbed vs. non-swabbed saliva using different extraction kits. Spearman correlation analysis comparing microbiome composition between swabbed and non-swabbed saliva samples. Panels **(A–D)** show the correlation between samples extracted using the BioChain kit following swabbing with one of four swab-based collection kits: ORACollect (swab1), Norgen Biotek (swab2), Omnigene.oral CORP (swab3), or ORAgene. DISCOVER (swab4), relative to their corresponding non-swabbed controls. Panels **(E–H)** display the same comparison using samples extracted with the Norgen kit, and panels **(I–L)** with the Qiagen kit. “Sal” refers to non-swabbed saliva samples. Red triangles indicate ASVs (amplicon sequence variants) found to be significantly different (*p* < 0.05) between swabbed and non-swabbed samples, as identified by DESeq2 analysis. This figure shows correlations between original and processed saliva samples, reflecting the impact of swabbing and storage.

**Figure 6:**
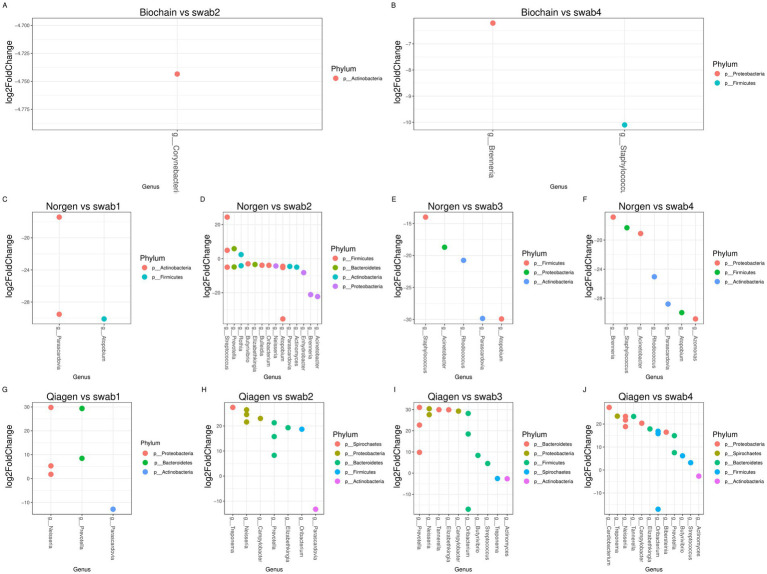
ASVs affected uniquely by each extraction and swabbing kit. **(A–B)** Log_2_ fold-change plots showing significantly altered ASVs between original (non-swabbed) saliva samples and swabbed saliva samples extracted using the BioChain kit. **(C–F)** Log_2_ fold-change plots for the same comparison using the Norgen extraction kit, and **(G–J)** using the Qiagen extraction kit. Positive values indicate ASVs that were more abundant in the original saliva samples. Plots are based on DESeq2 analysis; only ASVs with *p* < 0.05 and adjusted *p* < 0.09 are shown. This figure presents differential abundance analysis (DESeq2) comparing original and processed saliva samples.

**Figure 7:**
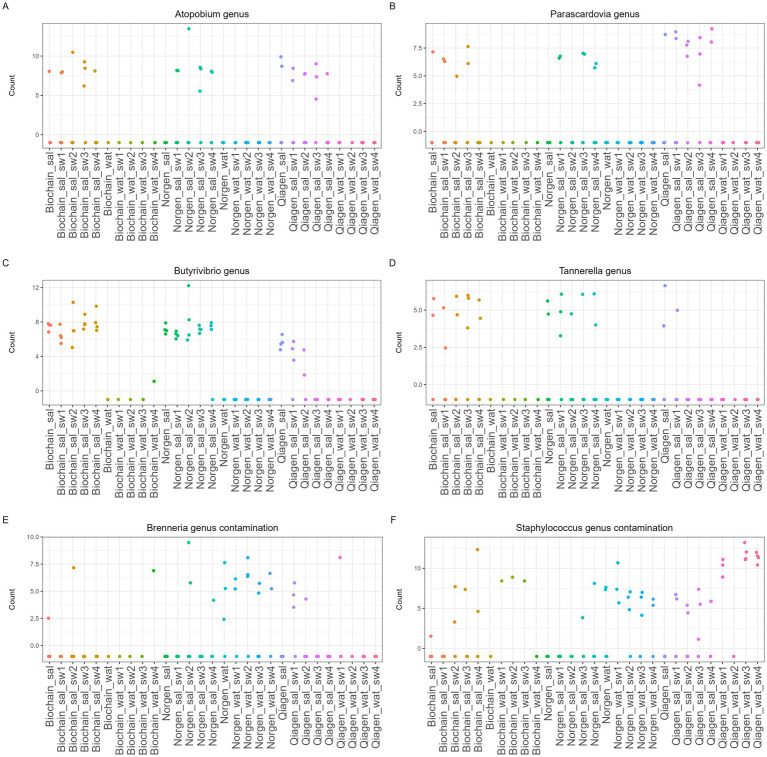
Specific ASVs affected uniquely by each extraction and swabbing kit. Logged bacterial counts of **(A)** *Aptopobium* genus, **(B)** *Parascardovia* genus, **(C)** *Butyrivibrio* genus, **(D)** *Tannerella* genus, **(E)** *Brenneria* genus, **(F)** *Staphylococcus* genus, in all samples. Plots generated from the DESeq2 analysis, ASVs presented are significantly altered ASVs, *p*-value < 0.05 corrected *p*-value < 0.09. Saliva (Sal), water (Wat), ORACollect (sw1), NORGEN BIOTEK (sw2), Omnigene.oral CORP (sw3), or ORAgene. DISCOVER (sw4). Each dot represents one sample. This figure highlights representative taxa that differ between original and processed samples.

To evaluate the effect of swabbing and storage on microbiome composition, we compared original saliva samples to processed samples using correlation and differential abundance analyses (Figures 5–6). We plotted the 16S count values between the original saliva samples and the same samples after they were swabbed and stored at room temperature for a week (Figures 3–7). We noticed that some ASVs were only relatively abundant following swabbing and others were only relatively abundant in the original saliva samples. Spearmen correlations revealed that saliva samples processed with the Biochain and Norgen kits correlated well with the original saliva (correlation ranging from 0.883 to 0.783 for biochain and from 0.888 to 0.862 for Norgen, Figures 5A–H). However, the saliva samples processed with the Qiagen kit exhibited lower correlation to the original saliva samples, with correlation scores ranging from 0.715 to 0.452 (Figures 5I–L).

We performed differential analysis using DESeq2 between the original and the processed saliva samples in order to determine whether specific bacteria were consistently being affected by the processing procedure. Differential abundance analysis was performed using DESeq2 with significance defined as adjusted *p*-value (Benjamini–Hochberg) < 0.05. DESeq2 identified three ASVs that were changed between the original and processed saliva samples with the Biochain kit (adjusted p-value < 0.05). The changes were observed in 2 different swabbing methods (Figures 6A,B). Moreover, DESeq2 analysis revealed that Norgen processing significantly altered 26 ASVs (Figures 7C–F, adjusted *p*-value < 0.05). Of these, 11 ASVs were detected at very low or undetectable relative abundance from the original or swabbed samples (placed on the X or Y axis, Figures 5E–H, in red). Most of these ASVs showed low or undetectable relative abundance from the original samples. Analyzing ASVs changed between swabbed and the original saliva extracted with the Qiagen kit revealed that 20 ASVs significantly changed (Figures 6G–J, adjusted *p*-value < 0.05). Of these, 17 ASVs showed low or undetectable relative abundance from the original or swabbed samples (Figures 5I–L, in red). Most of these ASVs had low or undetectable relative abundance from the swabbed samples.

We further plotted several ASVs that were significantly different in the DESeq2 analysis between the groups (Figure 7, adjusted *p*-value < 0.05). We demonstrate that the *Aptopobium* and the *Parascardovia* genus had low or undetectable relative abundance from the origin saliva sample extracted with the Norgen kit but increased relative abundance in the saliva samples after swabbing (Figures 7A,B, adjusted *p*-value < 0.05). Moreover, the *Butyrivibrio* and the *Tannerella* genus had higher relative abundance in all the origin saliva samples, but almost all swabbed samples extracted with a Qiagen kit had low or undetectable relative abundances (Figures 7C,D, adjusted *p*-value < 0.05). Furthermore, we demonstrate that the *Brenneria* and *Staphylococcus* genus had significantly increased relative abundance in the water samples compared to saliva samples (Figures 7E,F, adjusted *p*-value < 0.05).

## Discussion

In this study, we demonstrate that the choice of saliva collection and DNA extraction methods significantly impacts microbiome analysis and reproducibility. Our findings show that while the swabbing method has minimal influence on microbial composition, the DNA extraction kit has a profound effect. These observations were supported by PERMANOVA analysis. Additionally, we highlight that both swabbing and DNA extraction kits can introduce bacterial contaminants and lead to shifts in microbiome composition, which may have significant implications for clinical microbiome research, epidemiology, and microbiome study standardization.

Several studies have demonstrated that DNA extraction kits can introduce background contamination into 16S rRNA gene sequencing workflows (Drengenes et al., 2019; Shaffer et al., 2022; Salter et al., 2014), and computational approaches such as decontam have been developed to identify and remove contaminant sequences (Davis et al., 2018). However, most prior work has focused either on contamination arising from extraction kits alone or on downstream bioinformatic correction strategies. In contrast, the primary goal of this study was to evaluate how experimental factors, including sample collection methods, swab-based sampling, and short-term room temperature storage, influence microbiome profiles under conditions that reflect real-world sampling scenarios. Our results demonstrate that, regardless of the specific kit used, measurable microbial signals can arise in control samples following swabbing and storage. While computational approaches such as decontam are valuable, they depend on the availability and structure of appropriate controls and do not substitute for careful consideration of upstream methodological factors. Our study emphasizes the identification of experimental conditions that minimize contamination and best preserve the original biological signal. Together, our findings provide a practical framework for selecting sample-collection and processing methods that better reflect true oral microbiome composition in real-world settings.

This study systematically compared swab-based saliva collection kits and DNA extraction methods for saliva microbiome analysis while also evaluating the effects of extended incubation at room temperature. These factors are particularly relevant for at-home saliva collection and populations such as infants or individuals who cannot provide saliva spontaneously. Our work aims to facilitate high-quality, cost-effective sample collection with minimal participant burden. We tested four swab-based sampling kits and three column-based DNA extraction kits in all possible pairwise combinations. While previous studies have compared DNA extraction methods, they have often excluded swab-based saliva collection kits (Kuffel et al., 2024; Garbieri et al., 2017; Vesty et al., 2017) or evaluated swabs without specifically assessing their impact on the oral microbiome composition (Cascella et al., 2015; Deeley et al., 2016). Research on oral cavity DNA extraction has shown that different methods significantly influence bacterial community composition, impacting microbiome analysis (Rosenbaum et al., 2019). However, no prior studies have systematically assessed bacterial contamination introduced by these saliva DNA extraction kits, despite their potential to alter results (Thoendel et al., 2017). Unlike previous work, our study directly compares swab-based saliva collection kits and DNA extraction methods while evaluating bacterial contamination and shifts in microbiome composition after extended room temperature incubation. These findings provide crucial insights for optimizing saliva microbiome studies, particularly in at-home and clinical research settings.

Importantly, this study extends prior work by evaluating how multiple methodological factors, including sample collection strategy, storage conditions, and DNA extraction, interact to influence microbiome profiles under realistic conditions. Notably, the inclusion of swab-based collection methods addresses a key gap, as these approaches are widely used in populations unable to provide spontaneous saliva samples.

Our results show that samples extracted using the Qiagen kit exhibit distinct microbial profiles compared to those extracted with BioChain and Norgen kits (Supplementary Figures 1S, 2S). Specifically, Qiagen-extracted samples showed a higher relative abundance of *Firmicutes*, which could introduce bias in oral microbiome studies, given this phylum’s clinical relevance (Deo and Deshmukh, 2019). Similar findings have been reported in studies comparing DNA extraction kits for meconium samples, where the Qiagen microbiome kit demonstrated a bias toward *Firmicutes* (Stinson et al., 2018). However, in the absence of a known reference composition, it is not possible to determine whether this reflects overrepresentation by the Qiagen kit or underrepresentation by other kits. Therefore, these differences likely reflect methodological biases rather than true biological variation.

We also identified specific ASVs that significantly differed between original saliva samples and swabbed samples across extraction kits. Future studies should account for these ASVs and consider whether they result from extraction kit affinity, kit-associated contamination, or microbial overgrowth during incubation rather than genuine differences in the oral microbiome. For example, *Atopobium* and *Parascardovia* were below the detection threshold in fresh saliva samples extracted with the Norgen kit but increased relative abundance in swabbed samples, suggesting either an initial low abundance of these ASVs or bacterial proliferation post-swabbing. Similarly, *Butyrivibrio* and *Tannerella* were consistently detected in original saliva samples but were largely undetectable in swabbed samples extracted with the Qiagen kit. This discrepancy may be due to the swabbing method failing to collect these taxa efficiently or potential DNA loss during Qiagen kit processing. Additionally, incubation at room temperature for 1 week may have altered microbial composition, leading to the selective loss or overgrowth of certain bacteria.

Contamination is a critical concern in saliva microbiome studies due to the relatively low bacterial biomass compared to other niches, such as the gut (Deo and Deshmukh, 2019). In this study, amplification of bacterial DNA was detected in water-only negative controls, indicating low-level contamination originating from collection or extraction reagents. The extended one-week incubation period at room temperature may have further amplified the detectability of this contamination, either through bacterial proliferation or increased accessibility of contaminant DNA prior to downstream PCR analysis. We detected *Brenneria* and *Staphylococcus* in water samples, which should have been sterile due to UV treatment, indicating potential contamination from collection or extraction kits. Notably, Norgen-extracted samples exhibited the highest contamination levels in water controls, making this kit less suitable for salivary microbiome analysis. Of the three tested kits, BioChain provided the most reliable results, with minimal contamination and high microbial similarity between swabbed and non-swabbed samples, regardless of the swabbing method.

Moreover, we observed increased contamination in all kits following swabbing, likely due to bacterial proliferation during the one-week incubation period. This suggests that DNA preservation fluids in swabbing kits may not effectively inhibit bacterial growth over time. To mitigate this issue, we recommend UV treatment of collection tubes, swabs, and reagents, as well as the immediate addition of a lysis buffer after sample collection to expose DNA and prevent further bacterial growth. However, these approaches were not systematically validated in the current study and should be interpreted with caution.

From a practical standpoint, Norgen Biotek, which provides both swab-based sampling kits and DNA extraction kits, offered the most user-friendly options for sample collection, aligning with previous studies that favored this kit for ease of use (Deeley et al., 2016). However, technical ease should be balanced with microbiome integrity, as the Norgen DNA extraction kit exhibited significant contamination issues. Our findings align with previous work showing that DNA extraction kits introduce biases in microbial composition across body sites (Wright et al., 2023), emphasizing the need for careful selection of extraction methods in microbiome studies. Additionally, we found that the QIAamp DNA Microbiome (Qiagen) Kit required significantly longer processing time due to additional steps for host DNA removal, which may be a consideration for high-throughput studies.

This study has several limitations. First, the use of pooled saliva samples, while enabling controlled comparison of methodological factors, does not capture inter-individual variability of the oral microbiome and may limit generalizability to population-based settings. Second, although full-process negative controls were included, we did not incorporate dedicated extraction blanks or formal statistical contaminant identification approaches. In addition, the experimental design evaluated combined sampling, storage, and processing conditions rather than isolating individual factors, limiting mechanistic interpretation. Finally, the relatively small sample size may limit statistical power, and choices made to improve analytical stability, such as the use of a slightly relaxed adjusted *p*-value threshold, prevalence-based feature filtering, and rarefaction for alpha diversity, may reduce sensitivity to low-abundance taxa or introduce complementary biases and should be interpreted accordingly. In addition, taxonomic classification was performed using the Greengenes database, which may be less optimal for oral microbiome profiling compared to curated databases such as the Human Oral Microbiome Database (HOMD). However, as our analysis focuses on relative differences between conditions, this is unlikely to affect the overall conclusions.

Our study highlights that both saliva collection and DNA extraction methods are critical determinants in microbiome analysis. Commercially available kits introduce biases toward specific bacterial taxa and may contain contaminants that obscure true microbiome profiles. These methodological inconsistencies pose a significant challenge to reproducibility and comparability across studies. Standardization in microbiome research, improved reagents, and novel contamination removal strategies are essential for addressing these challenges and ensuring reliable results in future oral microbiome studies.

## Data Availability

The datasets presented in this study can be found in online repositories. The names of the repository/repositories and accession number(s) can be found at: https://www.ncbi.nlm.nih.gov/, PRJNA1420823.
