# Supplementary material for: Comparing saliva collection and DNA extraction methods for saliva-based microbiome profiling
Source: Front Microbiol. 2026 May 14;17:1809075. doi: 10.3389/fmicb.2026.1809075 (PMC13216038; doi:10.3389/fmicb.2026.1809075)
Supplement: Supplementary file 1 [file Data_Sheet_1.pdf]

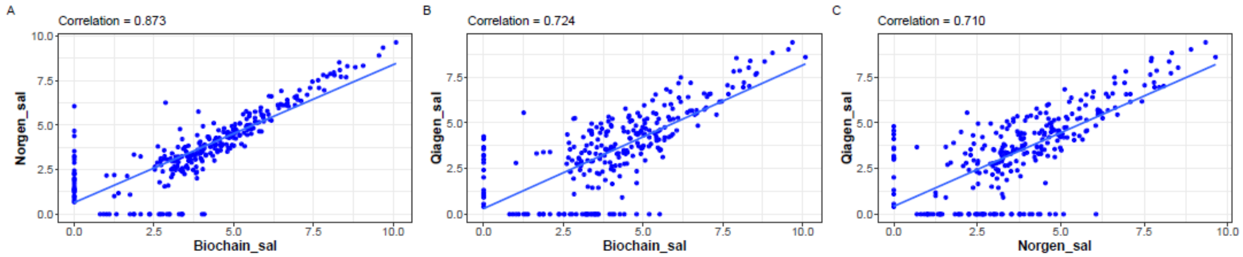

Figure 1S. Correlations of the microbiome composition of different extraction kits. Spearman correlation analysis of (A) correlation of the microbiome composition extracted using Biochain kit compared to Norgen. (B) correlation of the microbiome composition extracted using Biochain compared with Qiagen (C) correlation of the microbiome composition extracted using Norgen compared with Qiagen. Sal- Saliva.

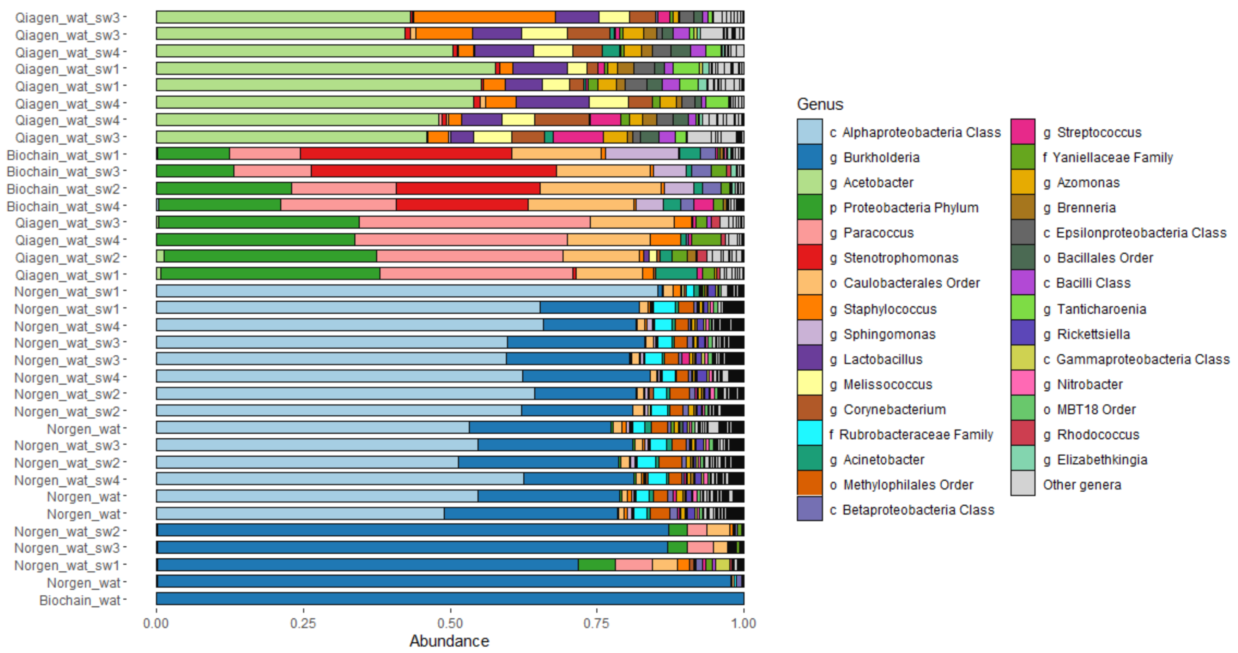

Figure 2S. Water contamination plot. Bar graph of taxa presented in swab and non-swabbed water samples extracted with Biochain, Norgen or Qiagen kits.
